# Mitochondria, calcium, and tumor suppressor Fus1: At the crossroad of cancer, inflammation, and autoimmunity

**DOI:** 10.18632/oncotarget.4537

**Published:** 2015-07-15

**Authors:** Roman Uzhachenko, Anil Shanker, Wendell G. Yarbrough, Alla V. Ivanova

**Affiliations:** ^1^ Department of Biochemistry and Cancer Biology, Meharry Medical College, Nashville, Tennessee, United States; ^2^ Vanderbilt-Ingram Comprehensive Cancer Center, Vanderbilt University, Nashville, Tennessee, United States; ^3^ Department of Surgery, Division of Otolaryngology, Yale School of Medicine, New Haven, Connecticut, United States; ^4^ Yale Cancer Center, Yale School of Medicine, New Haven, Connecticut, United States; ^5^ Department of Pathology, Yale School of Medicine, New Haven, Connecticut, United States

**Keywords:** Fus1/Tusc2, mitochondria, calcium, tumor suppressor, inflammation, autoimmunity

## Abstract

Mitochondria present a unique set of key intracellular functions such as ATP synthesis, production of reactive oxygen species (ROS) and Ca^2+^ buffering. Mitochondria both encode and decode Ca^2+^ signals and these interrelated functions have a direct impact on cell signaling and metabolism.

High proliferative potential is a key energy-demanding feature shared by cancer cells and activated T lymphocytes. Switch of a metabolic state mediated by alterations in mitochondrial homeostasis plays a fundamental role in maintenance of the proliferative state. Recent studies show that tumor suppressors have the ability to affect mitochondrial homeostasis controlling both cancer and autoimmunity. Herein, we discuss established and putative mechanisms of calcium–dependent regulation of both T cell and tumor cell activities. We use the mitochondrial protein Fus1 as a case of tumor suppressor that controls immune response and tumor growth via maintenance of mitochondrial homeostasis. We focus on the regulation of mitochondrial Ca^2+^ handling as a key function of Fus1 and highlight the mechanisms of a crosstalk between Ca^2+^ accumulation and mitochondrial homeostasis. Given the important role of Ca^2+^ signaling, mitochondrial Ca^2+^ transport and ROS production in the activation of NFAT and NF-κB transcription factors, we outline the importance of Fus1 activities in this context.

## INTRODUCTION

Mitochondria represent a vital signaling hub in cellular activities since they produce ATP, reactive oxygen species (ROS) and have the ability to shape intracellular Ca^2+^ signals [1, 2, 3]. Re-programming of mitochondrial metabolism and intracellular Ca^2+^ distribution are crucial for cells with high proliferative potential such as cancer cells and activated T lymphocytes. There are three main distinct features of mitochondrial metabolism common for tumor and activated T cells, which allow them to respond rapidly to the requirements associated with biomass increase:

1. *A switch from preferentially mitochondrial ATP production to aerobic glycolysis*. This phenomenon was first described by Otto von Warburg in 1956 for tumor cells and named after him as the Warburg effect. The Warburg effect was recently applied to explain metabolic reprogramming in activated T cells [4, 5, 6].
2. *Increased, but not excessive mitochondrial ROS (mitoROS) production* that is beneficial for tumor cells proliferation as well as for activation and proliferation of T lymphocytes [7–10, 11].
3. *Common metabolic pathways*. Similarly to tumor cells, activated T cells increase glutamine and glucose uptake while down-regulating free fatty acid beta-oxidation, a metabolic pathway important for resting and memory T cells. Glycolytic and glutaminolytic enzymes are key factors of the pentose-phosphate pathway involved in nucleotide synthesis. Further, glutamine catabolism fuels the tricarbonic cycle (TCA) via conversion into α-ketoglutarate as well as synthesis of various amino acids [6].

Therefore, mutations or altered levels of tumor suppressors or oncogenes that control mitochondrial homeostasis could adversely affect both proliferation of tumor cells and regulation of immune responses. In turn, impaired immune responses can promote cancer growth since the latter is preceded and maintained by chronic inflammation [12].

In this review, we will focus on the role of mitochondrial Ca^2+^ transport in the activation of T lymphocytes and proliferation of cancer cells. We will highlight the role of the mitochondrial tumor suppressor Fus1/Tusc2 that we recently characterized as a novel regulator of Ca^2+^ ion handling in mitochondria [12]. Using current knowledge about Fus1 functions, we will try to decipher how loss of Fus1 activities could promote development of autoimmune disorders and tumors observed in Fus1 knockout mice [13]. We believe that the described concept of a critical link between Ca^2+^ dynamics, mitochondrial metabolism and development of cancer, inflammation, and autoimmunity may be applied to other tumor suppressors and oncogenes providing new avenues for therapeutic interventions.

## FUS1 ACTIVITY AT THE CELLULAR AND WHOLE-ORGANISM LEVELS

### Fus1 as a tumor suppressor

*Fus1*, also known as *Tusc2* (tumor suppressor candidate 2), is a small (110 a.a.), highly conserved, ubiquitously expressed mitochondrial protein. Loss of the Fus1-harboring 3p21.3 chromosomal region or decrease in the Fus1 mRNA and protein levels have been reported in the majority of lung cancers [13, 14], mesotheliomas [15], bone and soft tissue sarcomas [16], and many head–and–neck cancers [17]. Noteworthy, Fus1 levels are decreased in bronchial squamous metaplastic and dysplastic lesions and even in normal epithelium of smokers [13, 14] suggesting that Fus1 deficiency in bronchial epithelial cells occurs at early stages of lung carcinogenesis and is associated with tobacco smoke. Tumor suppressor properties of Fus1 were established *in vitro* in lung cancer cell lines and *in vivo* in mouse lung cancer xenografts [18–20]. At the molecular level, the tumor suppressor activity of Fus1 is associated with the inhibition of tyrosine kinase c-Abl and activation of the Apaf-1 apoptotic pathway [21, 22, 23].

Recent data revealed multiple levels and mechanisms of Fus1 regulation. Suppression of Fus1 protein levels via miRNAs miR-93, miR-98, and miR-197 was suggested as one of potential mechanisms of malignization of bronchial epithelial cells [24]. Certain mRNA sequence elements in the 5′- and 3′-untranslated regions were identified as regulatory for Fus1 protein and mRNA expression [24, 25]. Partial methylation of Fus1 promoter region was identified in head and neck tumors and normal salivary rinses as compared to normal mucosa [17]. Remarkably, two Fus1/TUSC2 pseudogenes (TUSC2P) found on chromosomes X and Y that are homologous to the 3′-UTR of TUSC2 were described recently as regulators of Fus1 activities. [26].

Stage I clinical trials of lipoparticles that deliver Fus1 transgene to compensate for Fus1 deficiency in tumors of patients with chemotherapy refractory stage IV lung cancer [27] showed no significant side effects, resulted in the uptake and expression of the gene by primary and metastatic tumors, and produced anti-tumor effects [28]. Introduction of cationic liposomes with Fus1/hIL-12 co-expression plasmid led to reduction of lung tumor growth in mice [29]. Based on these trials, a new strategy that combines Fus1 lipoparticles and other agents (LKB1 plasmid, AKT inhibitor MK2206) or established chemotherapy agents have been proposed [30, 31, 23, 31].

Thus, Fus1 protein is a classical tumor suppressor that is decreased in tumor cells via multiple molecular mechanisms and, if restored, could produce a potent anti-tumor effect.

### Fus1 has a potential to control cancer and other pathologies via regulation of immune response and inflammation

It is commonly accepted that initial tumor growth often originates at sites with long-lasting chronic inflammation. Infiltration of immune cells to such sites creates specific inflammatory milieu (tumor-promoting cytokines, elevated ROS, etc.), which predisposes tissue cells to malignization and tumor growth [12, 32]. Chronic inflammatory processes affect all stages of tumor development [33]. *Fus1*-deficient mice display signs of chronic inflammation such as altered basal production of cytokines, NF-κB activation, and increased ROS levels in immune and epithelial cells [34]. Peritoneal exposure to asbestos, a carcinogenic mineral strongly linked to mesothelioma and lung cancer, resulted in a skewed cytokine response and more robust immune infiltration into the peritoneum of Fus1 KO mice [34]. Interestingly, in WT mice asbestos exposure led to down-regulation of Fus1 expression in cells of inflammatory milieu [34]. Our experiments with normal mesothelial cells of human origin exposed to asbestos revealed a ROS-dependent nature of Fus1 regulation [35] suggesting that ROS produced due to constant inflammation or environmental stress may keep Fus1 in epithelial and immune cells at a persistently low level. We also found that Fus1 knockdown in cancer cells confers resistance to oxidative stress [34].

Our *in silico* analysis of NCBI database GeoProfiles (http://www.ncbi.nlm.nih.gov/geoprofiles/) provided data on Fus1 expression in immune cells from different physiological and pathological states. We found that Fus1 is down-regulated in PBMC from multiple sclerosis patients (Ref # GDS3920) and in PBMC monocytes after interaction with bacteria Francisella tularensis (Ref # GDS3298). We also found that Fus1 level is decreasing during monocyte to macrophage differentiation. Interestingly, during subsequent macrophage polarization to M1 and M2, M1 macrophages showed 2-fold lower Fus1 level than M2 macrophages (Ref # GDS2429). At the same time, leukocytes and alveolar macrophages demonstrated up-regulation of Fus1 RNA upon activation with G-CSF (granulocyte colony-stimulating factor) (Ref # GDF2959) and LPS, respectively (Ref # GDS4419). Future experiments should further address dynamic changes of Fus1 expression in innate immune cells under polarizing conditions, during CD3/CD28 stimulation of T cells and Th1/Th2/Th17 polarization, during CD4+CD25+ Treg formation, etc.

At systemic level, Fus1 inactivation in mice led to development of multiple signs of autoimmunity reminiscent of systemic lupus erythematosus (SLE) [36]. Interestingly, adult Fus1 KO mice spontaneously developed tumors at the sites of chronic vessel inflammation, thus linking Fus1 loss to inflammation, autoimmunity, and tumorigenesis [36]. Thus, Fus1 as a suppressor of inflammation and carcinogenesis may serve as a therapeutic for treatment of inflammation-associated cancers, autoimmune and other inflammatory disorders.

### Putative molecular structure and cellular functions of Fus1

The molecular structure and functions of Fus1 protein are still not well established. Fus1 is a basic, soluble, globular protein residing in the mitochondria [36]. Early *in silico* studies of the Fus1 structure predicted a protein kinase A and C (PKA and PKC) phosphorylation sites (a.a. 43–57) as well as a site for ERK kinase binding (a.a. 33–47) [19, 37]. Also, computational and protein chip analyses revealed in Fus1 protein a C-terminally-located A kinase anchor protein (AKAP) interface, a PDZ (PSD95/Dlg1/ZO-1) class II domain, and interaction with PDZ class I of the pro-apoptotic protein Apaf-1 [19, 21, 38, 39]. We identified similarity of Fus1 with the DNA-binding domain of the IRF7 (Interferon Regulatory transcription Factor 7) [35]. Uno et al. demonstrated that Fus1 possesses a site for N-terminal myristoylation, which is crucial for tumor suppressor activities of Fus1 [19]. Finally, our recent pairwise alignment analysis revealed an EF-hand Ca^2+^-binding domain and a hydrophobic pocket with a potential capacity to bind the myristoyl tail in the Fus1 protein [40]. These features allowed to classify Fus1/Tusc2 as a member of Ca^2+^ /myristoyl switch protein family [41].

Fus1 activities related to cell functions are still not well characterized. Thus, cell cycle arrest and inhibition of lung cancer cell growth followed by Fus1 overexpression are consistent with the crucial role of Fus1 in cancer cell proliferation [18]. Synergism of Fus1 and p53 in the activation of the Apaf-1 apoptotic pathway suggests overlapping of the Fus1 and p53 pathways in the regulation of apoptosis in cancer cells [22]. Additionally, Fus1 displayed inhibitory effect toward tyrosine kinase c-Abl [21] and tyrosine kinase receptors, such as EGFR, PDGFR, and c-Kit [27], suggesting its crucial role in signal transduction. A recently established link between Fus1 and AMPK/AKT/mTOR signaling in cancer cells suggests involvement of Fus1 in cell survival and energetics [31]. However, molecular mechanisms of Fus1 involvement in these processes are still not determined.

### Fus1 and mitochondrial homeostasis

In our earlier work, we established Fus1 as a nucleus-encoded protein that resides in mitochondria [36]. Its mitochondrial residency was confirmed by our recent data on Fus1 loss-mediated mitochondrial dysfunction. Thus, we found that Fus1 loss in immune cells results in elevation of mitochondrial membrane potential (ΔΨm) at resting and asbestos-activated states. Additionally, asbestos-activated Fus1 KO leukocytes showed excessive ROS production and high levels of a mitochondrial protein UCP2 that regulates ΔΨm [34]. Further, depletion of Fus1 in cancer cells led to ΔΨm and ROS increases accompanied by resistance to oxidative stress [34]. Immune response to pulmonary infection by *Acinetobacter baumannii* in Fus1 KO mice also led to increased ΔΨm, ROS production, and UCP2 expression in innate immune cells that may mediate resistance of Fus1 KO mice to non-lethal bacterial infection [42].

Based on these data, we suggest that Fus1 plays a key role in the control of inflammation and cancer cell growth via maintenance of mitochondrial homeostasis. These Fus1 activities may mediate a crucial link between cancer and inflammation.

## MITOCHONDRIA IN CELLULAR CALCIUM HOMEOSTASIS AND CALCIUM SIGNALING

### Mitochondrial Ca^2+^ transport and its effect on mitochondrial homeostasis

**Figure 1:**
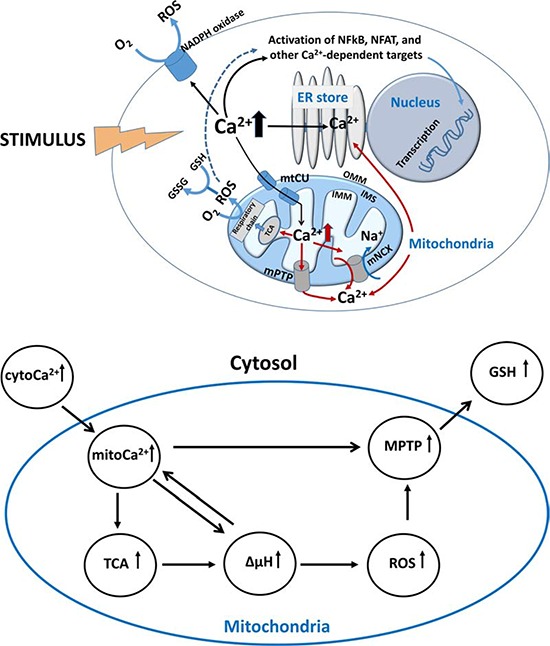
Ca2+/metabolic coupling in a cell **A.** *Ca^2+^ metabolism in response to cell stimulation*. Stimulus (TCR triggering, cytokines, mediators, hormones, etc.) leads to Ca^2+^ elevation in cytosol and activation of Ca^2+^-dependent targets including transcriptional factors NF-κB and NFAT, NADPH oxidase and other proteins. Important intracellular store for Ca^2+^ accumulation is endoplasmic reticulum (ER). After reaching threshold for Ca^2+^ concentration in cytosol, mitochondria begins taking up Ca^2+^ ions via Ca^2+^ uniport (mtCU) located in the inner mitochondrial membrane (IMM). Inside mitochondria, Ca^2+^ stimulates dehydrogenases of tricarbonic cycle (TCA) and H+ transport into intermembrane space (IMS) resulting in the increased respiratory chain activities, rise of electrochemical membrane potential (ΔΨm), and production of reactive oxygen species (ROS). In turn, ROS alter redox potential in mitochondria and other intracellular compartments by metabolizing reduced glutathione (GSH) to its oxidized form (GSSG). Finally, mitochondria use two basic mechanisms to extrude Ca^2+^: mitochondrial Na^+^/Ca^2+^ exchanger (mNCX) and mitochondrial permeability transition pore (mPTP). Lower panel. **B.** *Generalized scheme of Ca^2+^/metabolic coupling in mitochondria*.

Mitochondrial Ca^2+^ transport consists of two opposing processes: uptake and extrusion. Uptake of the positively charged Ca^2+^ ions is driven by a negative potential on the inner membrane (IMM), which is formed by pumping protons into the intermembrane space (IMS) driven by the mitochondrial respiratory chain [43] (Fig. 1). Ca^2+^ ions enter the mitochondrial matrix through a highly selective channels formed by a transmembrane IMM protein MCU (mitochondrial Ca^2+^ uniporter; also named MCUa) [44, 45] (Fig. 1). Another protein, MICU1 (mitochondrial Ca^2+^ uniporter 1) and its two paralogs, MICU2 and MICU3, were identified as Ca^2+^ sensors. They have two EF-hand motifs, which face IMS, interact with MCU, and control Ca^2+^ threshold and its import [44, 46, 45, 47, 48]. MCUR1 (mitochondrial calcium uniporter regulator 1), a yet another component of Ca^2+^ import machine, has been described recently as MCU-interacting, but the mechanism of its action remains unknown [49]. Another layer of complexity to the organization of the MCU complex (MCUC) was added after the identification of two novel components - MCUb, an inhibitor of MCUa, and EMRE (Essential MCU REgulator) that connects MCUa with the MICU1/2 regulatory subunits [reviewed in [50]]. Finally, the roles of ryanodine receptor RyR1, uncoupling proteins UCP2/3, and Ca^2+^/H^+^ exchanger LETM1 in Ca^2+^ uptake have also been demonstrated [51, 52, 53].

The directionality of Ca^2+^ ion transport, from the matrix towards the cytosol and vice versa, is maintained by Na^+^-dependent and -independent mechanisms. Na^+^-dependent export of Ca^2+^ is mediated via a mitochondrial Na^+^/Ca^2+^ exchanger (mNCX) represented by NCLX (alternative name is SLC8B1), which returns one Ca^2+^ ion back to the cytosol in exchange for three Na^+^ ions [54] (Fig. 1). mNCX is regulated by interactions with the number of proteins including protein kinases PKC and PINK1, SLP-2 and Bcl-2 [54, 55]. Finally, when Ca^2+^ concentration reaches the threshold, permeability transition pore (mPTP) provides mitochondria with Na^+^-independent Ca^2+^ extrusion from the matrix (Fig. 1). Since mitochondria possess high buffering capacity for Ca^2+^, mPTP prevents the formation of Ca^2+^ phosphate precipitates, which might destroy mitochondrial integrity. Thus, mitochondria employ several mechanisms for controlling Ca^2+^ levels, which are translated into signals for proliferation, differentiation, activation, etc.

### Metabolic coupling in mitochondria

**Figure 2:**
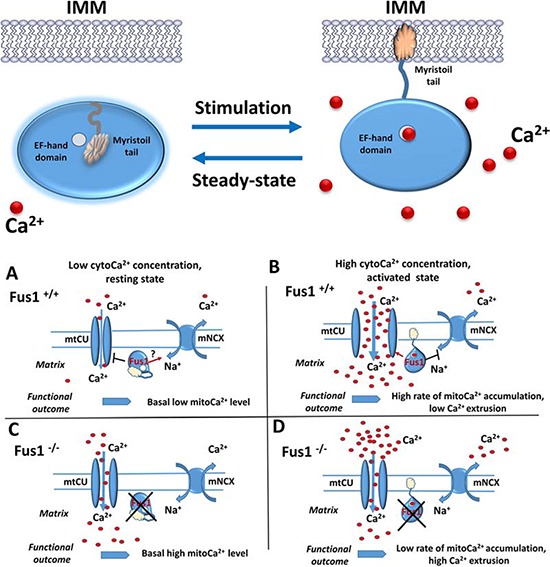
Hypothetic scheme of Fus1 functioning in mitochondria *Upper panel. Putative mechanism of molecular organization and activity of Fus1*. At Ca^2+^- free or low Ca^2+^ conditions (steady-state), EF-hand domain (open circle) of Fus1 protein is not occupied by Ca^2+^ ions while its myristoyl tail is folded inside the hydrophobic pocket of the protein preventing Fus1 from anchoring the membranes (left). Upon stimulation, elevation of Ca^2+^ in cytosol (red circles) results in Ca^2+^ ion binding to the EF-hand domain, release of myristoyl tail and anchoring of Fus1 protein to IMM. *Lower panel*. **A.** At Ca^2+^-free or low Ca^2+^ conditions, Fus1 may inhibit mtCU while simultaneously maintain the mNCX active state thus preventing accumulating Ca^2+^ in mitochondria. **B.** Elevation of Ca^2+^ leads to Fus1 activation and anchoring in IMM. Membrane tethering allows Fus1 to activate mtCU and inhibit mNCX that leads to retention of Ca^2+^ in mitochondrial matrix. **C.** When Fus1-mediated gatekeeping function is lost at a steady state, the mtCU channel is not tightly closed allowing cytosolic Ca^2+^ uptake while mNCX does not return Ca^2+^ ions back to cytosol. Thus, Ca^2+^ concentration in mitochondria of Fus1-deficient cells is higher. **D.** When Fus1-mediated gatekeeping function is absent during activation, the mtCU channel does not open to a full extent leading to insufficient buffering of cytosolic Ca^2+^ signals. Additionally, mNCX is over-activated resulting in extrusion of Ca^2+^ from mitochondria and an overall slow rate of mitochondrial Ca^2+^ accumulation.

Different metabolic processes are tightly coordinated and cross-regulated in mitochondria (Fig. 1A and 1B). After Ca^2+^ enters mitochondria, it activates key enzymes of the tricarbonic (TCA) cycle (pyruvate, isocitrate and alpha-ketoglutarate dehydrogenases) fueling the respiratory chain with protons and thereby increasing ΔΨm [56]. With the ΔΨm elevation, levels of ROS production in mitochondria are increased in a non-linear mode [57, 58]. Single-electron reduction of oxygen by complex I and III leads to the formation of superoxide anion followed by its conversion to hydrogen peroxide (H_2_O_2_) driven by superoxide dismutase 2 (MnSOD) [59]. Further, H_2_O_2_ is transported into the cytosol where it gets involved in redox signaling or used by the mitochondrial catalase in metabolic reaction leading to production of water. Glutathione peroxidase (GPX) supplies the catalase reaction with reductive equivalents in expense of reduced glutathione (GSH), which, in turn, forms oxidized dimers (GSSG). During the reverse reaction, a reduction of GSSG to GSH, glutathione reductase (GR) uses NADPH as a source of reductive equivalents. It leads to the oxidation of NADH catalyzed by transhydrogenase (TH) resulting in decrease of charge on IMM (ΔΨm depolarization) since protons are directed towards the reduction of NAD+ for TH reaction but not for the formation of ΔΨm [60]. mNCX becomes activated after primary accumulation of Ca^2+^ in the mitochondrial matrix to prevent Ca^2+^ overload and the induction of apoptosis [61, 62, 63]. Thus, mNCX limits histamine-induced alterations in mitoCa^2+^ accumulation and production of NADP(H) by extrusion of Ca^2+^ from mitochondria [64]. However, excessive mNCX-mediated Ca^2+^ efflux could lead to deficiency in the activation of respiratory chain complexes, NAD/NADH disbalance, and drop in ATP synthesis [65, 66]. Due to inappropriate TCA cycle activation and diminished NADPH formation, it results in ROS overproduction [67, 68]. A generalized scheme of Ca^2+/^metabolic coupling is presented in Fig. 2B.

It is important to note that the levels of mitochondrial Ca^2+^, ROS, ΔΨm, and thiols define activities of mitochondrial channels such as inner membrane anion channel (IMAC) and mPTP, thereby defining cell fate [60, 69].

Overall, Ca^2+^ accumulation in mitochondria coordinates multiple coupled processes into cross-regulatory transduction loops and, thus, allows connecting metabolic demands with cell fate (apoptosis, proliferation, etc.).

### Calcium-mediated cross-talk between mitochondria and ER

Currently, it is accepted that mitochondria shape intracellular Ca^2+^ signals, affect transcription of nuclear-encoded genes, and regulate multiple cellular functions [70, 71]. The mechanisms of mitochondrial involvement include the control of store-operated Ca^2+^ entry (SOCE) and cross-talk between mitochondria and endoplasmic reticulum (ER), a key calcium storage organelle [1, 2, 3]. SOCE represents a multi-step cascade of events that start from depletion of Ca^2+^ from ER induced by agonist stimulation that triggers Ca^2+^ entry across the plasma membrane. This process is tightly associated with the IP3 (inositol-3-phosphate) production that leads to the release of Ca^2+^ from the ER store. The decrease of ER Ca^2+^ levels is accompanied by dissociation of Ca^2+^ ions from EF-hands of STIM1 protein. This results in STIM1 conformational changes, its redistribution towards the plasma membrane, and interaction with an integral plasma membrane protein ORAI1, a pore subunit of Ca^2+^ Release-Activated Ca^2+^ (CRAC) channel. The interaction between ORAI1 and STIM1 maintains trans-membrane Ca^2+^ currents through CRAC resulting in the rise of free intracellular Ca^2+^. It was established that the magnitude of Ca^2+^ currents is strictly dependent on the strength of the ORAI1/STIM1 interaction, which is increased proportionally to the level of ER Ca^2+^ decrease. Upon Ca^2+^ elevations, CRAC and IP3 receptors are subjected to slow inactivation thus forming a negative regulatory loop [72, 73].

The involvement of mitochondria in SOCE includes buffering of Ca^2+^ released from IP3–induced ER store thereby promoting emptying ER stores and extended opening of CRAC channels. Moreover, due to the close proximity of mitochondria to the plasma membrane and ER stores, Ca^2+^ uptake by mitochondria reduces Ca^2+^-dependent slow inactivation of CRAC and IP3 receptors [2, 3].

Since mitochondria and ER form multiple contacts, the cross-talk between these two organelles plays an important role in the maintenance of overall Ca^2+^ homeostasis [74, 3] that occurs via several ways:

1. After initial uptake of Ca^2+^ mitochondria slowly release it through mNCX in the closest proximity to the ER Ca^2+^ ATPase pump (SERCA) thus refilling ER Ca^2+^ store.
2. Mitochondria-produced ROS oxidize proteins involved in Ca^2+^ transport and thus activate ryanodine (Ry) and inositol triphosphate (IP3) receptors involved in Ca^2+^ release and inhibit Ca^2+^ ATPases involved in Ca^2+^ uptake.
3. Mitochondria produce ATP that supplies SERCA and plasma membrane Ca^2+^ ATP pumps with energy.

Recently, the key role of MCU in SOCE-mediated Ca^2+^ transport has been confirmed. In particular, it was shown that MCU and UCP2 are required for decreasing inactivation of IP3 receptors and oligomerization and redistribution of STIM1, thus supporting store-operated Ca^2+^ entry [75, 76].

### Role of mitochondria in cellular calcium oscillation

The first cytosolic Ca^2+^ spike determines generation of oscillatory Ca^2+^ signals in cytosol [77]. Being increased after cell activation, cytosolic Ca^2+^ is accumulated in mitochondria through Ca^2+^ uptake mechanisms. Following a drop in cytosolic Ca^2+^ concentration, mNCX releases Ca^2+^ ions from mitochondria that, due to the close proximity to ER, leads to sensitization of ryanodine and inositol triphosphate receptors and triggers subsequent (so-called regenerative) Ca^2+^ release from ER store. Cyclic repeats of Ca^2+^ shuttling between cytoplasmic, ER, and mitochondrial compartments form a pattern of Ca^2+^ oscillations [77]. The role of mitochondria in calcium oscillations has been shown for the number of cells including HeLa [77, 78], cardiomyocytes [79], neurons [80], astrocytes [81], etc. Significant role of mNCX was demonstrated for ER Ca^2+^ refilling and ER/mitochondria Ca^2+^ recycling in B-lymphocytes after B cell receptor activation [82]. The role of modulation of Ca^2+^ oscillations in the regulation of transcription factors’ activation is highlighted in the next chapter.

### Global Ca^2+^ signals, mitochondrial Ca^2+^ accumulation, and regulation of transcription

Changes of cytosolic Ca^2+^ in response to cell activation are organized as oscillations with periodic drops and rises of Ca^2+^ [83, 84]. In 1997, it was established that frequency of Ca^2+^ oscillations determines the activities of NFAT and NF-κB, two major transcriptional factors important for T cell activation [85]. High-frequency Ca^2+^ oscillations with 6–7 min periodicity produce stimulatory effect on NFAT since its activation is linked with periodic cycles of phosphorylation-dephosphorylation by calcineurin [86]. On the contrary, the activity of NF-κB requires low-frequency Ca^2+^ signals since its activation is regulated by the degradation and re-synthesis of the protein inhibitor subunit IkB that takes a longer time span (about 30 min.) [87].

As described above, mitochondria regulate cellular Ca^2+^ oscillations in a cell type-specific manner. Thus, inhibition of mNCX with CGP37157 in HeLa cells leads to a switch of agonist-induced Ca^2+^ oscillations from high- to low-frequency pattern. At the same time, in fibroblasts with spontaneous Ca^2+^ oscillatory activity, CGP37157 enhances Ca^2+^ oscillation frequency [78]. This goes in line with the observation made on sensory neurons where impairment of trans-mitochondrial Ca^2+^ transport (this term refers to the combination of mitochondrial Ca^2+^ import and export) resulted in the elimination of intracellular Ca^2+^ plateau and diminished nuclear import of NFAT [80]. Altered Ca^2+^ accumulation in cells due to defects in trans-mitochondrial Ca^2+^ transport also leads to dephosphorylation of IkB and activation of NF-κB as well as ATF2 and the NF-κB-dependent HIF-1a axis, all of which have pro-tumorigenic activities [88]. Another example of decoding the frequency of Ca^2+^ oscillations is the modulation of Ca^2+^/calmodulin (CaM)-dependent kinase CaMKII activation. Higher-frequency Ca^2+^ oscillations or low-frequency spikes of long duration increase this enzyme activity through CaM trapping and, thus, maintain CaMKII autophosphorylation [89, 90]. Recently, Samanta et al. demonstrated that a pore-forming subunit of mtCU, MCU, is involved in the generation of Ca^2+^ oscillations evoked by physiological stimuli [91]. In particular, MCU maintains Ca^2+^ oscillatory pattern through sustained regenerative Ca^2+^ release and SOCE-mediated Ca^2+^ entry as well as via decrease in CRAC channel and IP3 receptor inactivation. As an outcome, knockdown of MCU leads to decrease in the activity of c-Fos and NFAT transcription factors [91]. Another mechanism of mitoCa^2+^ transport involvement in transcriptional regulation and metabolism has been described recently. Sustained mitochondrial Ca^2+^ loading regulates NAD+/NADH metabolism via TCA activation. Marcu et al. demonstrated that changes in the mitochondrial NAD+/NADH ratio can acutely influence protein acetylation by modulating both the activity and expression of sirtuins not only in mitochondria but also in the cytosolic and nuclear compartments [92].

### Fus1 as a putative new mitochondrial Ca^2+^ sensor protein

Coordinated changes in ΔΨm and ROS production at the basal and activated states in immune cells with Fus1 KO prompted us to seek for common origin of these changes. Levels of ΔΨm and ROS are both dependent on the mitochondrial Ca^2+^ content [43, 93]. Using multiple protein alignment, we predicted that Fus1 has an EF-hand Ca^2+^ -binding domain and a hydrophobic pocket for interaction with the myristoyl tail [40], a saturated fatty acid covalently attached to the N-terminal glycine residue of the Fus1 protein [19]. This irreversible post-translational protein modification is called myristoylation. The similarity of the Fus1 protein structure to the one of the members of the Ca^2+^/myristoyl switch family allowed us to hypothesize that Fus1 is a potential Ca^2+^ sensor (Fig. 2, upper panel). The mechanism of action for Ca^2+^/myristoyl switch proteins was described about 20 years ago [94, 95, 41]. The proteins of Ca^2+^/myristoyl switch family have two main conformations (Fig. 2, upper panel). At a steady-state Ca^2+^ concentration, the myristoyl tail is clamped by aromatic residues inside the hydrophobic pocket. This conformation ensures inactive state of the proteins. When Ca^2+^ concentration raise (i.e., due to cell activation), Ca^2+^ ions bind to the EF-hand resulting in release of the myristoyl tail that anchors the protein to the membrane via intercalation into lipid bilayers (Fig. 2, upper panel). This mechanism helps to localize proteins to distinct signaling compartments, which is critical for coordinated regulation of signaling cascades [94, 41]. Recently, several myristoylated proteins residing in mitochondria have been described. Among them are proteins of the inner and outer mitochondrial membranes: GTPase ARL4D [96], ChChd3 [97], NADH-cytochrome b5 reductase [98, 99], truncated forms of actin [100], and Bid [101]. However, Fus1 is the first putative member of the Ca^2+^/myristoyl switch protein family localized to mitochondria. A distinct feature of the Fus1 protein structure is an overlap between the EF-hand (54–65 a.a.) and myristoyl-binding (45–110 a.a.) domains [40]. Proteins of the Ca^2+^/myristoyl switch family usually have these two regulatory elements separated from one another by a large amino acid stretch [41, 95]. However, in the ancient, evolutionary conserved Fus1 protein, Ca^2+^-binding motif (the EF-hand) resides inside the myristoyl-binding pocket [40].

The theoretically predicted Ca^2+^ sensor properties of Fus1 have been confirmed by measuring mitochondrial Ca^2+^ uptake in Fus1-deficient cells (Fig. 2). Depending on Ca^2+^ level in cytosol, Fus1 promoted a dual effect on the Ca^2+^ transport into mitochondria. At steady-state conditions, Fus1 maintained mitoCa^2+^ level by preventing its further uptake by mitochondria (Fig. 2, lower panel, A). However, during Ca^2+^ elevation in the cytosol induced by calcium agonist ionomycin or CD3/CD28 activation, Fus1 allowed accumulation of Ca^2+^ in mitochondria [34] (Fig. 2, lower panel, B). Thus, Fus1 belongs to a growing list of regulators of mitochondrial Ca^2+^ uptake identified in the last several years (MICU1–3, MCUR1 etc.) [102, 12].

We propose that being a Ca^2+^ sensor, Fus1 regulates a threshold for mitochondrial Ca^2+^ uptake. Indeed, basal Ca^2+^ levels in mitochondria of Fus1 KO cells were on average 2-fold higher than in WT cells (Fig. 2, lower panel, C). However, upon challenge with high-level Ca^2+^ pulses, mitochondria of Fus1 KO cells showed delayed and low-amplitude Ca^2+^ accumulations (Fig. 2, lower panel, D) [40]. Thus, *Fus1* loss results in inefficient translation of cytosolic Ca^2+^ elevations into mitochondrial Ca^2+^ rises [40]. In this respect, Fus1 is similar to MICU1, a mitochondrial EF-hand Ca^2+^ sensor that interacts with the MCU pore subunits. MICU1 plays a gatekeeping role at low cytosolic Ca^2+^ concentrations while mediates an uptake of Ca^2+^ ions by mitochondria after stimulation with Ca^2+^-mobilizing agents [46, 103, 104, 105].

Cells possess a broad set of different Ca^2+^-binding EF-hand proteins for decoding Ca^2+^ signals. Each EF-hand protein has its own activation threshold that depends on the number and affinity of Ca^2+^-binding motifs. Thus, it is predicted that proteins containing only one such motif become activated earlier in response to Ca^2+^ changes than proteins with two or more Ca^2+^-binding domains, since occupation of more than one site with Ca^2+^ requires higher ion concentration [90]. Future comparative studies will determine if Fus1 that possesses a single Ca^2+^-binding domain [40] responds to calcium rises faster than proteins with multiple EF-hand domains.

Based on our experimental data [40], we suggest that one of the possible mechanisms of Fus1 involvement in Ca^2+^ regulation is inhibition of mNCX, a mitochondrial membrane antiporter protein that removes one Ca^2+^ ion from mitochondria in exchange for three Na^+^ ions and thus protects from excessive Ca^2+^ accumulation in mitochondria [54]. Noteworthy, similarly to Fus1 KO cells, MICU1 loss also results in the enhanced activity of mNCX [46]. In addition to mNCX regulation, Fus1 most likely employs other mechanisms of calcium control since mNCX inhibition by CGP37157 only partially restored accumulation of Ca^2+^ in mitochondria of Fus1^−/−^ cells [40].

Taken together, these data point at the interaction of the described Ca^2+^-binding proteins with multiple targets aimed at the fine-tuning of mitochondrial Ca^2+^ transport through modulation of its uptake and extrusion. We hypothesize that this process might include several steps and involve mtCU activation and mNCX inhibition depending on the amplitude, frequency, and other parameters of Ca^2+^ response.

## ROLES OF MITOCHONDRIA, CALCIUM, AND FUS1 IN TUMORIGENESIS AND IMMUNITY

### Mitochondrial Ca^2+^ transport and tumorigenesis

Changes in intracellular Ca^2+^ concentration are used as a universal signaling mechanism to control diverse biological processes including proliferation and cell death, two hallmarks of tumorigenesis. Perturbation in the mechanisms of mitochondrial Ca^2+^ transport, e.g. via changes in the mtCU or mNCX activities, are translated into changes in signal transduction cascades as well as activities of transcription factors and their targets. Therefore, initial pro-tumorigenic events in cells as well as tumor cell fate are mediated via changes in intracellular calcium. Thus, it was shown that elevated cytosolic Ca^2+^ concentration increased activation of ERK1/2 and PKC that led to the activation of pro-tumorigenic transcription program [88]. Recently, LETM1, an EF-hand containing Ca^2+^/H^+^ transporter, was shown to control proliferation by shaping cellular bioenergetics via mitochondrial Ca^2+^ flux. Loss of LETM1 enhanced mitoROS production, AMPK (AMP activated kinase) activation, and promoted autophagy and cell cycle arrest [106].

Tumor cell survival was shown to be regulated by mitochondrial calcium fluxes [107]. Thus, aggressively growing malignant melanoma cells showed accelerated mitochondrial Ca^2+^ fluxes, which coincided with enhanced SOCE-mediated Ca^2+^ influx and high levels of constitutively active protein kinase B/Akt (PKB). Interruption of trans-mitochondrial Ca^2+^ transport abolished SOCE-mediated Ca^2+^ influx, inactivated PKB, slowed cell growth, and increased sensitivity to apoptosis [107].

The importance of NADPH oxidase (NOX) for tumor growth have been broadly demonstrated [108, 109]. The activity of NOX, which produces ROS and controls transcription of a number of genes, was shown to be dependent on mitochondrial calcium uniporter (mtCU). Suppression of mtCU leads to retention of Ca^2+^ in the cytosol and over-stimulation of PKC, which is necessary for phosphorylation of NADPH subunits and its activity [110].

The important role of mitochondria and Ca^2+^ in apoptosis makes mitochondrial Ca^2+^ transport an attractive target for designing novel therapeutic approaches for cancer treatment [111]. Indeed, some tumor suppressors and oncogenes promote their activities via modulation of mitochondrial Ca^2+^ uptake and, *vice versa*, established regulators of mitoCa^2+^ level demonstrated their involvement in carcinogenesis. Thus, pro-oncogenic constitutively active STAT3 protein inhibits expression of nuclear-encoded genes of the mitochondrial electron transport chain (ETC) thereby decreasing ΔΨm, a driving force for Ca^2+^ uptake, and, therefore, resulting in the decrease of mitoCa^2+^ levels [112]. Recently, it was shown that pro-tumorigenic, aberrantly high expression of miR-25 or its family members miR-92a and miR-363 selectively decreases calcium uniporter (MCU) levels and mitochondrial Ca^2+^ uptake that results ultimately in resistance to apoptosis and increased cancer cell survival [111, 113]. Furthermore, it was also shown that in the absence of MICU1, an essential regulator of mitochondrial Ca^2+^ threshold, mitochondria become constitutively loaded with Ca^2+^ triggering excessive ROS generation and increasing sensitivity to apoptotic stress [104]. Thus, MICU1 was characterized as a key molecule through which cancer cells acquire resistance to toxic effects of positively charged gold nanoparticles (+)AuNPs. Silencing of MICU1 allowed to overcome resistance to (+)AuNPs initiating the mitochondrial pathway for apoptosis [114]. Thus, modulation of mitochondrial Ca^2+^ levels by tumor suppressors and oncogenes is an important mechanism in cancer cell survival and apoptosis that could be targeted in anti-tumor therapeutic approaches.

### Mitochondrial Ca^2+^ transport and T cell activation

Upon activation, T cells interact with professional antigen-presenting cells and form immune synapses (IS) with the latter. At the IS, the energy-consuming processes of ion transport take place. To meet energy demands, T cell activation is linked to mitochondrial translocation into the areas of the IS [115, 116] where mitochondria provide local ATP production and Ca^2+^ buffering. Local Ca^2+^ buffering at the IS facilitates larger and more sustained Ca^2+^ influx and concomitant activation of transcription factors NFAT, AP1, and NF-κB [115].

Ca^2+^ influx immobilizes mitochondria at the plasma membrane through interaction with the mitochondrial calcium-binding protein Miro, which is needed for kinesin-1-dependent mitochondrial transport mediated via an adaptor protein Milton [117, 118].

It was proposed that at the IS, increase in mitochondrial ATP synthesis occurs via mtCU-dependent activation of the TCA cycle [68]. Recently, a new mtCU-dependent mechanism of T cell activation was described by Ledderose et al. [119]. Immediately after activation, T lymphocytes rapidly increase ATP production in response to mtCU-mediated mitoCa^2+^ elevation. Consequently, ATP is translocated to the extracellular space by a hemi-channel Panx1 transporter where it binds to and activates membrane P2X purinergic receptors. These events help maintaining Ca^2+^ signaling in T cells [119].

Different signaling events during T cell activation are dependent on changes in mitochondrial Ca^2+^ levels. Thus, T cells activation requires production of mitochondrial ROS, which is induced by TCR-initiated calcium flux into mitochondria after mtCU opening that leads to increased complex III turnover [10, 111].

Major T helper cell subsets, Th1 and Th2, are distinct in their intracellular calcium handling. Thus, Th2 cells have lower activity of mtCU and mtCU-mediated CRAC currents and, therefore, slower Ca^2+^ influx. This explains a lower level of Th2 activation as compared to Th1 cells upon identical activating stimuli and production of different sets of cytokines [112].

### Fus1-mediated calcium handling in the NFAT and NF-κB activation

NFAT and NF-κB are two major transcription factors (TFs) that drive the T cell activation program including expression of IL-2, IL-4, IL-6, IL-12, TNFa, IFNg, CD40L, FasL, etc [120, 121]. In our study, Fus1 KO CD4^+^ T cells showed elevated basal expression of NFAT and NF-κB target genes while the activation index for these genes (a fold difference in RNA expression before and after stimulation) was decreased as compared to WT CD4+ T cells [40]. These data point at aberrant activation of the NFAT and NF-κB pathways in Fus1–deficient T cells that may stem from alterations in mitochondrial Ca^2+^ accumulation, mitochondrial ROS production [9, 10], as well as mtCU and mNCX activities [80, 10, 91]. As mentioned above, in cancer cells increase in cytosolic Ca^2+^ leads to the activation of NFAT and NF-κB and, thus, to stimulation of a pro-tumorigenic gene network [88]. Prevention of the cytosolic Ca^2+^ uptake by mitochondria enhances resistance to apoptotic stimuli. On the contrary, excessive accumulation of mitoCa^2+^ via inhibition of mNCX results in cell death [62]. We showed that in normal epithelial cells, *Fus1* loss alters accumulation of Ca^2+^ in mitochondria. Thus, in Fus1 KO cells mitoCa^2+^ level was elevated at basal conditions while at Ca^2+^-loading conditions it was decreased as compared to WT cells [40]. These changes in mitochondrial Ca^2+^ concentration correlated with chronic activation of NF-κB-dependent genes and unbalanced inflammatory response, thus making Fus1-deficient cells prone to malignant transformation and growth [40, 36].

### How tumor suppressor proteins, mitochondrial calcium, anti-microbial response and autoimmunity are linked?

Fus1 is a typical tumor suppressor [18–20, 21, 22, 23] with a newly assigned function of a regulator of mitochondrial calcium handling [40]. For many years, roles of tumor suppressors and oncogenes in the immune system regulation have remained underappreciated. However, recent findings implicated many of these proteins in autoimmune disorders, while immuno-regulatory proteins (STAT3, STAT5, NF-κB, NFAT etc.) have been linked to tumorigenesis [122, 123, 124, 125, 126]. Mechanistically, many of these proteins execute their functions via modulation of Ca^2+^ signaling [127]. Thus, Bcl-2 protein, a member of the Bcl-2 family (Bcl-2, Bcl-xL, Bax, Mcl-1) that serves as a main barrier against autoimmune disorders [128], modulates calcium signaling via mtCU potentiation [129]. Anti-apoptotic Bcl-2 is also capable to suppress mtCU by raising the threshold for Ca^2+^ uptake in HEK293 cells [130, 131]. Another member of the Bcl-2 protein family, Mcl-1, was shown to play a key role in hematological and biliary malignancies as well as in fatal autoimmune disorders. Mcl-1 inhibits Ca^2+^ accumulation in mitochondria and prevents apoptosis, most likely through binding to p32, a positive regulator of mtCU [132, 133]. Mcl-1 plays a critical role in survival of Treg cells while other anti-apoptotic proteins, Bcl-2 and Bcl-xL, are dispensable for this process [134]. However, the importance of cooperation between Bcl-xL and transcription factor FoxP3 was demonstrated for development and persistence of Treg cells preventing arthritis [135]. Bcl-xL was found to be involved in the activation-induced cell death (AICD) initiated in T cells after TCR activation [136, 137]. Mechanistically, Bcl-xL interacts with VDAC promoting transport of Ca^2+^ from the cytosol to mitochondrial matrix and, thus, modulating calcium signaling [138]. In the absence of Bim, a tumor suppressor from the Bcl-2 family, T cells display impaired Ca^2+^ response and NFAT activation after TCR ligation due to prevalence of inhibitory interaction of Bcl-2 with IP3 receptor on the surface of ER. As a result, mice with targeted inactivation of Bim in hematopoietic system do not develop experimental autoimmune encephalomyelitis and induced diabetes [139].

Classical tumor suppressor p53 was shown to control Treg cell differentiation and, thus, protect against systemic autoimmune inflammation [140–142]. In cancer cells, p53 induces expression of a novel calcium channel TRPC6 and recruits it for Ca^2+^ release-dependent drug-induced apoptosis [143]. On the other hand, prevention of p53 transport to the nucleus and its re-localization to mitochondria lead to decreased accumulation of Ca^2+^ in mitochondria [144].

We showed that Fus1 KO mice spontaneously develop systemic lupus erythematosus, an autoimmune disease [36]. Ca^2+^ dynamics and associated signaling pathways are dramatically altered in autoimmune disorders [145–148]. Human peripheral blood lupus T cells are characterized by decreased NF-κB activation, elevated levels of ΔΨm, mitoCa^2+^ and cytoCa^2+^ after TCR activation [149]. It was reported that in inflammatory bowel disease, T cells from inflamed tissues have increased cytoCa^2+^ levels [150]. At the same time, importance of a proportional NF-κB activation was demonstrated in rheumatoid arthritis: enhanced TNFα-induced NF-κB activation led to diminished Ca^2+^ signaling but prolonged secretion of inflammatory cytokines [151]. Recently, the role of ORAI1 in autoimmunity has been established. Mouse T cells lacking SOCE Ca^2+^ current promoted via ORAI1 failed to induce colitis after adoptive transfer into lymphopenic mice [152]. Thus, organization of Ca^2+^ signaling in T cells meets demands of the complex systems controlled by different multi-level checkpoints to prevent pathological changes.

**Figure 3:**
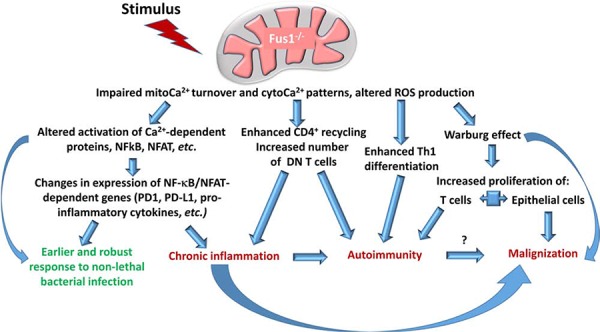
Role of Fus1 in the control of immune response and tumor growth Stimulus (TCR triggering, cytokines, mediators, hormones, etc.) applied to *Fus1−/−* cells results in altered Ca^2+^ response due to impaired mitoCa^2+^ accumulation that, in turn, alters ROS response. These changes have several critical outcomes and include enhanced Th1 differentiation, changes in activity of NFkB and NFAT transcription factors, and potential predisposition to a profound metabolic switch (Warburg-like effect) providing these cells advantage toward proliferation. Taken together, it may lead to development of pathological systemic conditions (red font) such as chronic inflammation, autoimmune syndrome, or tumor growth. At the same time, these changes in immune cells may be advantageous (green font) allowing more effectively combat bacterial infections.

Regulatory T cells can suppress immune cell activities and, therefore, are important for evasion of tumor cells from immune surveillance [12]. Thus, a direct inhibitory effect on T lymphocytes or antigen-presenting cells was demonstrated for CD4+CD25+FoxP3+ Treg cells [153]. In our study, we found that in an asbestos-induced inflammatory environment favorable to tumor growth, Fus1 loss led to excessive amount of CD3^+^CD4^−^CD8^−^ T cells called double-negative (DN) T cells that were accumulated locally in peritoneum [34]. Although the nature of DN T cells origin is not well defined, our observation of early down-regulation of surface CD4 molecule after activation of Fus1 KO CD4^+^ T cells [40] suggested that they could originate from CD4^+^ and CD8^+^ T cells after loss of the surface localization of CD4 or CD8 co-receptors, which is in-line with other studies [154]. Interestingly, patients with SLE, an autoimmune disease that Fus1 KO mice are prone to, have a significantly larger pool of circulated DN T cells [155]. Moreover, CD4^+^ T cells from SLE patients have decreased levels of surface CD4 and TCRzeta receptors due to increased activity of the HRES-1/Rab4 system involved in the endocytosis of cell surface molecules and regulated by mTOR [156, 157]. mTOR is regulated by multiple mechanisms and pathways including calcium signaling [158, 159, 160]. Reducing cytosolic calcium inhibits phosphorylation of S6K1, an mTOR effector [161]. In our study, bacteria-infected Fus1 KO lung tissues had higher levels of activated S6 protein, a downstream target of mTOR [42]. Mechanistically, lower mitochondrial Ca^2+^ and, as a result, higher cytoplasmic Ca^2+^ in Fus1 KO cells may activate mTOR and potentially promote differentiation of T cells towards the DN phenotype (Fig. 3). As a consequence, DN T cells could inhibit local immune response leading to the switch from acute to chronic disease course, which is conducive to cancer growth.

Th1 and CD8^+^ T cells represent hallmark subsets of autoimmunity since IL-2 and IFNy promote CD8^+^ T cell response directed towards parenchymal cells [162]. We showed that *Fus1* deficiency leads to the shift in Th differentiation towards Th1 [40] that supports our early results on the autoimmune phenotype in Fus1 knockout mice [36]. The role of mitochondria in Th polarization is not well studied. However, it was shown that Th2 cells have more effective mechanisms of Ca^2+^ clearance from the cytosol while in Th1 cells cytosolic Ca^2+^ oscillate for the longer period of time after activation [163]. Furthermore, elevated ROS levels support Th2 polarization [164]. Thus, decreased mitochondrial Ca^2+^ accumulation, subsequent retention of Ca^2+^ in the cytosol, and decreased ROS production after stimulation in Fus1 KO T cells might promote Th1 stemming (Fig. 3). As a result, it will be accompanied by excessive production of IFN-gamma and tissue inflammation. We also cannot rule out that the autoimmune response induced by Fus1 loss may predispose to initiation of tumor growth since autoimmune inflammation has a chronic course.

Immune responses and autoimmunity are tightly controlled via expression of immunosuppressive/immunoregulatory molecules such as CTLA-4, PD-1/PD-L1, and BTLA that, in turn, are regulated via activation of transcription factors NFAT, NF-κB, STAT3 or STAT4 [165, 166, 167]. We demonstrated that *Fus1* loss leads to a decreased PD-1 and PD-L1 expression in CD3/CD28-activated CD4^+^ T cells, which correlates with the impaired activation of NFAT/NF-κB-mediated transcription (Fig. 3) [40]. Thus, changes in the levels of immunoregulatory molecules may promote the autoimmune phenotype in Fus1 KO mice (Fig. 3). At the same time, we speculate that similar changes in the innate immune cells can enhance their protective potential. Indeed, PD-1-deficient dendritic cells demonstrated significantly improved protective response against *Listeria* [168].

In our study, we showed that *Fus1* KO mice have increased resistance to *A. baumannii* pneumonia mediated by prompt and enhanced innate immune response to bacterial infection stemming from early activation of proinflammatory pathways (Fig. 3) [42]. The innate immune response is mediated by dendritic cells, neutrophils, macrophages, natural killers and some other cell types. Carrithers et al. demonstrated that in macrophages sodium channel NaV1.5 and sodium-calcium exchanger mNCX form a coupled system, which controls the generation of prolonged Ca^2+^ oscillations in the periphagosomal region important for uptake of mycobacteria [169]. Another molecular complex with important implication for anti-microbial defense is NADPH oxidase [170]. NADPH oxidase is sensitive to intracellular Ca^2+^ concentration since full activity of the enzyme requires stimulation of calcium-regulated PKC [171]. This signaling axis is controlled by mitochondrial Ca^2+^ uptake in a way that slower uptake results in retention of Ca^2+^ in cytosol and up-regulation of NADPH oxidase activity [110]. Loss of early effective Ca^2+^ sequestration of by mitochondria may result in increased activation of Ca^2+^-dependent enzymes such as NADPH oxidase, which is necessary in particular for activation of NLRP3, a component of inflammasome [172].

*Fus1*-mediated mitochondrial dysfunction may bring pro-tumorigenic changes not only to immune but also to epithelial cells (Fig. 3). Indeed, as we discussed above, *Fus1* deficiency leads to a diminished mitochondrial Ca^2+^ uptake and, therefore, increased levels of Ca^2+^ in cytosol of epithelial cells, which could increase pro-survival potential of these cells as was shown for cancer cells [114, 104]. Moreover, since Ca^2+^ is involved in the activation of key TCA cycle enzymes, we assume that inefficient Ca^2+^ accumulation in mitochondria would reduce TCA cycle turnover, thus promoting switch to glycolytic metabolism (Warburg effect) (Fig. 3) [4–6]. Noteworthy, the same metabolic switch is crucial for activated T cells since glycolysis provides necessary level of NADPH equivalents, ATP and interplays with pentose-phosphate cycle fueling cells with monomers for nucleic acid, proteins, and lipid synthesis [4–6]. Thus, if *Fus1*-deficiency supports the same metabolic process in activated CD4^+^ T lymphocytes, it would result in chronic activation or permanent pre-activated T cells state as well as in triggering the avoiding mechanisms associated with immune tolerance (Fig. 3).

## CONCLUDING REMARKS

Recent data suggest that many oncogenes, tumor suppressors, and immune regulators modulate mitochondrial homeostasis to perform their key activities. Calcium, a ubiquitous second messenger, is involved in a broad spectrum of physiological events. Remodeling of Ca^2+^ homeostasis that triggers changes in ROS and ATP production, calcium signaling and metabolic state is mediated through mitochondria and ER, two major intracellular calcium stores. Currently, there is as yet limited understanding of the role of specific modulators of Ca^2+^ metabolism/homeostasis/transport in controlling tumorigenesis and immune response. The mitochondrial protein Fus1, a tumor suppressor and immunoregulator highlighted in this review, is only “the tip of the iceberg” of a whole new concept on the key role of the calcium-regulating machinery in cancer and immunity [173, 174]. There is a long road ahead to determine the entire network maintaining and altering calcium homeostasis in a cell and assign a specific role to each of the Ca^2+^ regulators in cancer and immunity. Future pre-clinical studies should focus on designing/testing the potential agents (small molecules-, DNA-, or protein-based) that selectively target cancer- or immune cell-specific Ca^2+^ influx/efflux pathways and may serve as a basis for developing novel therapeutic interventions. Mitochondrial pharmacology nowadays is a fast developing field, which offers multiple tools to modulate mitochondrial processes (including Ca^2+^ transport, ROS production, etc.). These tools may be combined with classical schemes of chemo- or immunotherapy for maximal efficiency.
